# Structural and Functional Perspectives of Optineurin in Autophagy, Immune Signaling, and Cancer

**DOI:** 10.3390/cells14221746

**Published:** 2025-11-07

**Authors:** Gianluca Medigovic, Hari Krishnareddy Rachamala, Shamit Kumar Dutta, Krishnendu Pal

**Affiliations:** 1 College of Liberal Arts and Sciences, University of Florida, 330 Newell Dr, Gainesville, FL 32603, USA; gianluca5050@gmail.com; 2 Department of Biochemistry and Molecular Biology, Mayo Clinic Florida, 4500 San Pablo Road S, Jacksonville, FL 32224, USA; rachamala.hari@mayo.edu (H.K.R.); dutta.shamit@mayo.edu (S.K.D.)

**Keywords:** optineurin, protein–protein interaction, autophagy, cancer, immune signaling

## Abstract

Optineurin (OPTN) is a multifunctional adaptor protein that regulates diverse cellular processes, including inflammatory signaling, autophagy, vesicular trafficking, and immune responses. This multifaceted role of OPTN is made possible by the presence of a complex structure comprising multiple domains that interact with different proteins to exert various functions important for modulating key signaling processes. Mutations in OPTN are linked with several human pathologies including glaucoma, Paget’s disease of bone, Crohn’s disease, and neurodegenerative diseases such as amyotrophic lateral sclerosis, and dementia. Emerging evidence suggests that OPTN has a complex and context-dependent role in cancer biology as well. It is upregulated in pancreatic ductal adenocarcinoma and hepatocellular carcinoma but downregulated in lung and colorectal cancers, indicating its dual role as a potential oncogene or tumor suppressor depending on the cellular environment. Additionally, OPTN plays a critical role in preventing immune evasion in colorectal cancer by maintaining interferon-gamma receptor 1 (IFNGR1) expression and supporting dendritic cell-mediated T-cell priming, thereby enhancing antitumor immune responses. Despite its significance in oncogenic pathways and immune regulation, the therapeutic potential of targeting OPTN in cancer remains largely unexplored. This review aims to provide a comprehensive understanding of OPTN’s pleiotropic functions, highlighting its role in autophagy, inflammation, immune surveillance, and cancer progression. By elucidating its diverse regulatory mechanisms, we seek to encourage further research into the therapeutic implications of OPTN in cancer treatment and immunotherapy.

## 1. Introduction

Optineurin (OPTN) is a multifunctional adaptor protein that plays a crucial role in various cellular processes, including autophagy, inflammation, and immune signaling. It has a molecular weight of approximately 66 kDa and is encoded on chromosome 10p13. Comprising 577 amino acids, OPTN is highly expressed in the brain, heart, and liver, suggesting its significance in maintaining homeostasis in vital organs [1]. Structurally, OPTN contains several functional domains, including a ubiquitin binding domain (UBD), a NF-κB essential modulator (NEMO)-like ubiquitin binding in ABIN and NEMO (UBAN) domain, and a Microtubule Associated Protein 1 Light Chain 3 (LC3) interacting region (LIR) domain, facilitating its interactions with multiple cellular proteins and regulatory pathways [2]. Recent studies have increasingly linked OPTN to both tumor-suppressive and oncogenic functions, demonstrating its dual role in cancer biology. On one hand, OPTN has been shown to regulate NF-κB signaling by interacting with Receptor Interacting Protein 1 (RIP1), thereby suppressing excessive inflammatory responses that contribute to tumor progression [3]. It also plays a vital role in selective autophagy by partnering with key autophagy receptors, such as SQSTM1, and ubiquitin ligases like Homologous to the E6-AP Carboxyl Terminus (HECT) domain and ankyrin repeat containing E3 ubiquitin–protein ligase 1 (HACE1), to facilitate the clearance of damaged organelles and misfolded proteins, thereby preventing oncogenic transformation [4]. Additionally, OPTN regulates vesicular trafficking through its interactions with Rab GTPases and Myosin VI, ensuring proper cellular transport mechanisms that are critical for maintaining intracellular balance [5]. Conversely, OPTN has also been implicated in cancer progression, with its expression levels varying across different malignancies. It is upregulated in pancreatic ductal adenocarcinoma (PDAC) and hepatocellular carcinoma (HCC), potentially contributing to tumor growth and survival [6,7]. In contrast, its downregulation in lung and colorectal cancers (CRC) suggests a tumor-suppressive function, reinforcing the complexity of its role in oncogenesis [4,8]. Moreover, in CRC, OPTN is essential for immune surveillance by preventing immune evasion mechanisms and IFNGR1 stability, which enhances T-cell priming by dendritic cells [8,9]. Given its multifaceted roles in autophagy, cellular signaling, and immune regulation, OPTN emerges as a compelling target for therapeutic intervention in cancer. However, despite its growing recognition as a key player in oncogenesis, only a few studies have explored its potential as a druggable target. This review aims to provide a comprehensive examination of the structural and functional aspects of OPTN in cancer, highlighting its diverse roles across autophagy, tumorigenesis, and immune modulation. By summarizing the latest findings and identifying knowledge gaps, this review underscores the rationale for considering OPTN as a potential therapeutic target and encourages future research to explore its clinical relevance in cancer treatment.

## 2. Structure of OPTN

OPTN is a structurally complex adaptor protein composed of multiple functional domains including the coiled-coil domain, leucine zipper, LIR and the specific ubiquitin binding region known as the UBAN domain, each of which contributes to OPTN’s function as a molecular scaffold in different cellular pathways [2].

N-Terminal domains: The N-terminus of OPTN contains coiled-coil motifs that play a crucial role in protein–protein interaction. One of the most well-characterized functions of the N-terminal coiled-coil domain is its binding to TRAF Family Member Associated NF-κB Activator (TANK) binding kinase 1 (TBK1), which phosphorylates OPTN and enhances its LC3 and ubiquitin-binding affinity [10]. This phosphorylation is essential for OPTN’s role in selective autophagy and immune signaling [11,12]. Additionally, the adjacent leucine-zipper motif to the coiled-coil region serves as a binding platform for the small GTPases Rab8 and Rab12, which are critical for vesicular trafficking [5,13].

LIR domain: The LIR domain of OPTN enables direct binding to LC3 proteins, which are crucial components of the autophagy pathway. This interaction ensures the recruitment of OPTN to LC3-positive autophagic vesicles, where it functions as an autophagy receptor [2]. By bridging ubiquitinated cargo with LC3 puncta, the LIR domain plays an indispensable role in selective autophagy, allowing for the efficient degradation of intracellular pathogens, protein aggregates, and dysfunctional organelles.

UBAN domain: OPTN’s ubiquitin binding domain, UBAN, is involved in the binding with actin-based motor protein Myosin VI, which regulates OPTN’s spatiotemporal functions of autophagy and secretory vesicle fusion [2,14]. The UBAN domain, located within the central region of OPTN, is structurally similar to the UBAN domain of NEMO, allowing it to participate in key inflammatory signaling pathways [14]. This domain is responsible for indirect binding to RIP1, a key mediator of necroptosis and NF-κB signaling [15]. RIP1 will bind to OPTN via its UBAN domain interacting directly with deubiquitinating enzyme CYLD (Cylindromatosis) and interleukin-1 receptor-associated kinase 1 (IRAK1), positioning OPTN as a regulatory molecule in inflammatory and immune responses [16]. Through these interactions, OPTN helps modulate inflammation by controlling ubiquitination events that affect signaling cascades.

C-Terminal domains: The C-terminus of OPTN harbors additional zinc-finger domains and leucine-zipper motifs, which contribute to protein stability and interaction specificity. The UBD within this region plays a key role in recognizing and binding to polyubiquitinated cargo, which is essential for its function in autophagy [17]. Through its UBD, OPTN interacts with the ubiquitin ligase HACE1, the autophagy receptor SQSTM1, and ubiquitinated organelles marked for degradation [2,4]. These interactions facilitate the recruitment of autophagic machinery, ensuring the clearance of damaged mitochondria and misfolded proteins to maintain cellular homeostasis [4].

The coordination of these domains allows OPTN to act as a key player in autophagy, inflammation, and immune signaling. Dysfunction in OPTN structure, whether through mutations or altered expression levels, has been implicated in various diseases, including neurodegenerative disorders and cancer [6,7,8]. Understanding the structural–functional relationships of OPTN provides valuable insights into its role in cellular homeostasis and highlights its potential as a therapeutic target in disease contexts.

## 3. OPTN in Various Protein–Protein Interactions

OPTN’s role in autophagy is largely mediated by its interactions with TBK1, HACE1, and SQSTM1 [4,10]. The presence of multiple UBDs allows OPTN to recognize and bind to polyubiquitinated cargo, facilitating the degradation of damaged organelles, protein aggregates, and intracellular pathogens [18]. OPTN preferentially binds K63-linked polyubiquitin chains, which are indicative of autophagic degradation rather than proteasomal degradation [19]. Additionally, zinc-finger domains within OPTN enhance its ability to bind ubiquitinated substrates [18]. The LIR works alongside the UBD to position OPTN as a bridge between ubiquitinated cargo and autophagosomes [18]. This facilitates autophagosome maturation and fusion with lysosomes.

TBK1 serves as a key regulatory kinase for OPTN in autophagy. Phosphorylation of OPTN by TBK1 enhances its affinity for polyubiquitinated cargo, thereby improving its efficiency in selective autophagy. Phosphorylation at Ser-177 and Ser-473 by TBK1 enables tighter binding to ubiquitin chains and enhances OPTN’s recruitment to autophagosome formation sites [10]. On the other hand, OPTN recruits TBK1 for its autophosphorylation and function, while TBK1 phosphorylates OPTN, improving its ability to clear damaged mitochondria [12]. Without TBK1, OPTN’s oligomerization potential and autophagic efficiency are significantly reduced [20]. OPTN plays a critical role in Type I interferon (IFN) signaling by regulating TBK1-dependent phosphorylation of Interferon Regulatory Factors (IRF3 and IRF7). OPTN facilitates TBK1 accumulation, indirectly aiding the phosphorylation of IRF3 and IRF7, leading to an enhanced Type I IFN response [11]. Studies in OPTN-deficient mice have shown that reduced TBK1 activity leads to lower IRF3 phosphorylation, causing decreased IFN-β levels, impairing antiviral responses [21].

The E3 ubiquitin ligase HACE1 plays a crucial role in OPTN’s function by linking it to ubiquitin chains and autophagy receptors. HACE1 ubiquitinates Lys-193 of OPTN with K48-linked polyubiquitin chains. While K48-linkages usually signal proteasomal degradation, in this case, they promote autophagic degradation and the formation of LC3 puncta [4,22]. HACE1 also promotes the SQSTM1-OPTN complex, which enhances autophagy by guiding autophagosomes to their targets. SQSTM1 is required for this axis to function, preventing the accumulation of dysfunctional autophagy receptors and mitigating oxidative stress-related damage [4].

OPTN plays a regulatory role in NF-κB signaling through its interactions with RIP1 and CYLD, influencing inflammation and necroptosis. OPTN shares homology with NEMO, allowing it to compete for binding to RIP1 and interfere with the IKK complex, thereby inhibiting NF-κB activation. OPTN recruits CYLD, a deubiquitinating enzyme, to cleave K63-linked ubiquitin chains from RIP1, preventing NF-κB translocation to the nucleus and suppressing inflammatory gene expression [3,14]. OPTN binds IRAK1, inhibiting the polyubiquitination of TRAF6, thereby preventing IL-1β and Toll-like receptor (TLR)-induced NF-κB activation [16]. In T cells, OPTN acts as a negative regulator of T-cell receptor (TCR)-induced NF-κB activation [23]. OPTN modulates Receptor-Interacting Protein Kinase 1 (RIPK1) turnover, suppressing necroptotic cell death. OPTN depletion in mice increases sensitivity to necroptosis, further highlighting its protective role in inflammation-associated cell death [15].

OPTN plays an integral role in post-Golgi trafficking, membrane transport, and lysosomal degradation by interacting with Rab8, Rab12, and Myosin VI. Rab8 facilitates exocytosis from the Golgi, stabilizing vesicle formation [5]. Rab12 regulates autophagy receptor recycling, ensuring receptor availability for subsequent degradation cycles [24]. Myosin VI is an ATP-dependent motor protein that moves vesicles along actin filaments. Rab8, Rab12, and Myosin VI all bind to OPTN’s coiled-coil domain, while Rab8 also binds its leucine-zipper and LIR domains and Myosin VI interacts with the UBD. OPTN colocalizes with Rab8 and Myosin VI at the Golgi, maintaining Golgi integrity and vesicle trafficking [13]. Without OPTN, Myosin VI fails to localize to the Golgi, leading to fragmentation and disruptions in vesicle formation and trafficking [25].

**Figure 1:**
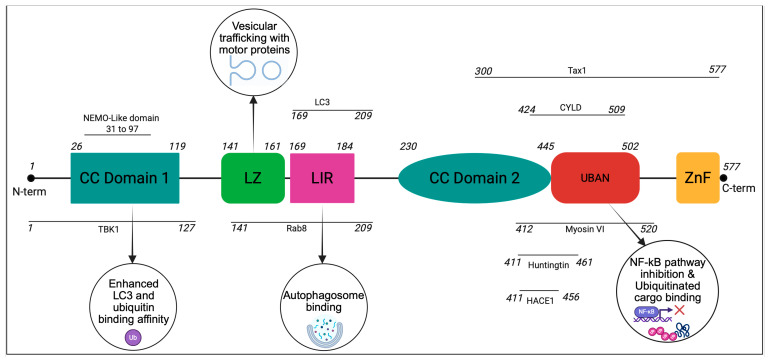
Key binding partners and functions of OPTN structural domains: OPTN is mainly composed of coiled-coil (CC) domains, a leucine zipper (LZ), an LC3-interacting region (LIR), a UBAN domain, and a zinc finger (ZnF) domain at its C-terminus. The interaction sites of OPTN with its key binding partners are also shown. Connections to the phagophore are primarily mediated by the LIR, while ubiquitinated cargo utilizes the ubiquitin binding regions. Coiled-coil motifs and leucine zippers provide additional binding opportunities with other interacting partners.

Taken together, OPTN’s extensive protein–protein interaction position it as a multifunctional regulatory protein that integrates autophagy, immune signaling, inflammation, and vesicular transport (Figure 1). Through its ability to interact with TBK1, HACE1, SQSTM1, RIP1, CYLD, Rab GTPases, and Myosin VI, OPTN ensures cellular homeostasis by coordinating degradation pathways, inflammatory responses, and intracellular trafficking. Given its role in these critical processes, dysregulation of OPTN has been linked to various diseases, including neurodegenerative disorders, cancer, and inflammatory conditions, making it an important target for further research and therapeutic intervention.

## 4. The Role of OPTN in Autophagy

OPTN is involved in macroautophagy, a cellular recycling mechanism that captures and degrades ubiquitinated proteins and organelles via lysosomal processing [26]. Macroautophagy, which relies on the sequestration of cellular components within autophagosomes, is regulated in part by the ATG12-ATG5-ATG16L1 complex, which facilitates lipidation of LC3 to LC3-II, an essential step in autophagosome formation [27]. This complex is recruited by the interaction of OPTN with WD repeat domain phosphoinositide-interacting protein 2 (WIPI2) [27]. In the absence of OPTN, the recruitment of the ATG12-5-16L1 complex to WIPI2 is compromised, reducing LC3 lipidation and impairing autophagy progression [27]. Within the realm of cellular surveillance, OPTN is already a known regulator of xenophagy, a selective form of macroautophagy that focuses on the removal of invading pathogens and viruses [28]. For instance, single and double knockout zebrafish models reveal how OPTN deficient mutants were more susceptible to infection, especially when knocked out in parallel with SQSTM1 [29]. Beyond its role in xenophagy, OPTN has also been linked to neuroprotective autophagy, accelerating the clearance of phosphorylated Tau (p-Tau) aggregates, and preventing Tau-related neurodegenerative pathology. In vitro and in vivo, OPTN expression decreased p-Tau levels, leading to increased learning and memory in mice models [30]. Many of the same mechanisms are employed maliciously and protectively in the context of cancer.

## 5. OPTN in Autophagy-Linked Cancer

**Figure 2:**
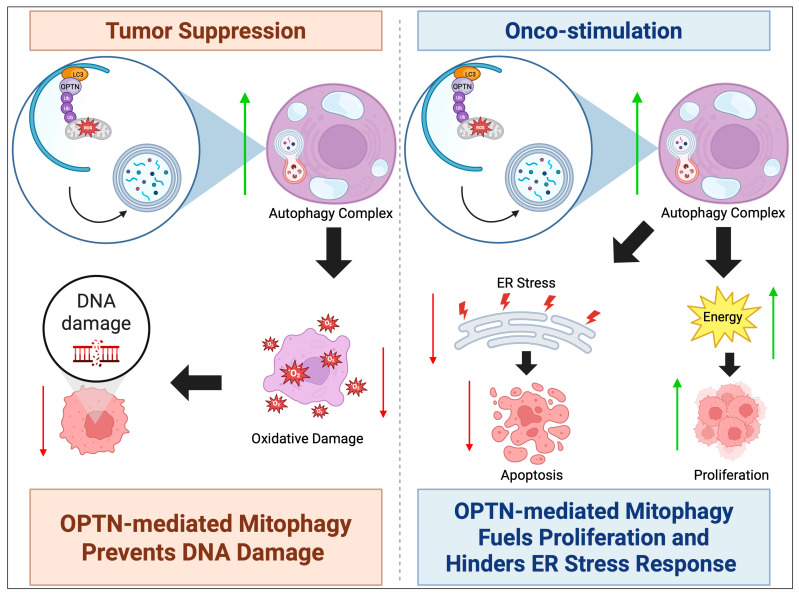
OPTN can either act as a tumor suppressor or oncogene. In autophagy, OPTN’s tumor suppressor mechanism commonly involves upregulating mitophagy to suppress ROS levels and oxidative damage, preventing DNA damage-related tumorigenesis. The mechanism of onco-stimulation involves OPTN’s upregulation of mitophagy providing fuel for mature carcinomas to proliferate, in addition to suppressing ROS-induced ER stress that would trigger apoptosis. An upward green arrow indicates upregulation, whereas a downward red arrow denotes downregulation.

The role of OPTN in cancer is highly context-dependent, functioning either as a tumor suppressor or as an oncogenic driver. Given its integral role in autophagy, OPTN can either support tumorigenesis by fueling cancer cell metabolism or act as a suppressor by mitigating cellular stress and oxidative damage (Figure 2).

Onco-Stimulatory Role of OPTN in Cancer: In certain malignancies, overexpression of OPTN contributes to tumor growth and survival by enhancing autophagy and metabolic adaptation. This is particularly evident in PDAC, where OPTN is highly upregulated, correlating with poor patient prognosis [6]. Knockdown of OPTN in PDAC cell lines leads to endoplasmic reticulum (ER) stress activation and apoptosis, accompanied by an increase in chaperone-mediated autophagy (CMA), suggesting that cancer cells rely on alternative autophagy pathways when macroautophagy is disrupted [31]. The induction of ER stress may also upregulate protein chaperones involved in CMA, or alternatively, may activate p53-mediated apoptosis [32]. Additionally, OPTN depletion in PDAC cells results in cell cycle arrest at the S phase and G1 checkpoint, as indicated by elevated levels of non-phosphorylated cyclin D1 and reduced expression of Cyclin-Dependent Kinase 6 (CDK6) [31]. This arrest prevents cellular replication and growth, reinforcing the link between OPTN overexpression and PDAC cell survival.

Furthermore, cancer cells exhibiting high OPTN levels preferentially engage in mitophagy, selectively degrading mitochondria to maintain energy production and reduce oxidative stress [33]. Mitophagy is typically initiated when PTEN-induced putative kinase 1 (PINK1) accumulates on the outer mitochondrial membrane of damaged mitochondria. In normal conditions, PINK1 is rapidly degraded upon mitochondrial import, but in dysfunctional mitochondria, it phosphorylates and activates E3 ubiquitin ligase Parkin, which then recruits OPTN to initiate mitophagy [34]. This process is essential in limiting reactive oxygen species (ROS) production, thereby preventing oxidative DNA damage. However, in HCC, OPTN overexpression leads to excessive mitophagy, significantly increasing ATP production through beta-oxidation while suppressing ROS levels and inhibiting the ER stress response. Studies have demonstrated that beta-oxidation byproducts in wild-type HCC cells are ten times higher than in OPTN knockout HCC cells, while ROS levels are markedly increased in the latter. Consequently, tumor progression is significantly reduced in OPTN-deficient HCC cells due to diminished mitophagy. Similarly, engineered overexpression of OPTN in liver cancer cells accelerates proliferation, reinforcing its role as an oncogenic driver [7].

Tumor-Suppressive Role of OPTN in Cancer: In other contexts, low OPTN expression is associated with increased tumorigenesis, highlighting its tumor-suppressive properties. In lung cancer, where OPTN is frequently downregulated, there is a strong correlation between high OPTN levels and improved relapse-free survival. One of the key mechanisms underlying OPTN’s tumor-suppressive role involves its interaction with HACE1, an E3 ubiquitin ligase that facilitates OPTN’s role in autophagic flux regulation. The OPTN-HACE1 axis plays a critical role in lung cancer suppression by promoting selective autophagy. HACE1 ubiquitinates autophagic cargo, marking it for degradation and facilitating its recognition by OPTN [4]. This interaction initiates autophagy and promotes the recruitment of the SQSTM1 complex, which accelerates the degradation process. The successful removal of damaged organelles via autophagy reduces ROS accumulation, thereby minimizing oxidative DNA damage and limiting cancer cell proliferation [8]. Indeed, overexpression of OPTN in lung cancer cell lines results in a 63% reduction in proliferation, while in vivo studies show that tumors in OPTN-HACE1-expressing cells are five times smaller than in cells lacking these proteins [4].

## 6. OPTN in Cancer Immunity

**Figure 3:**
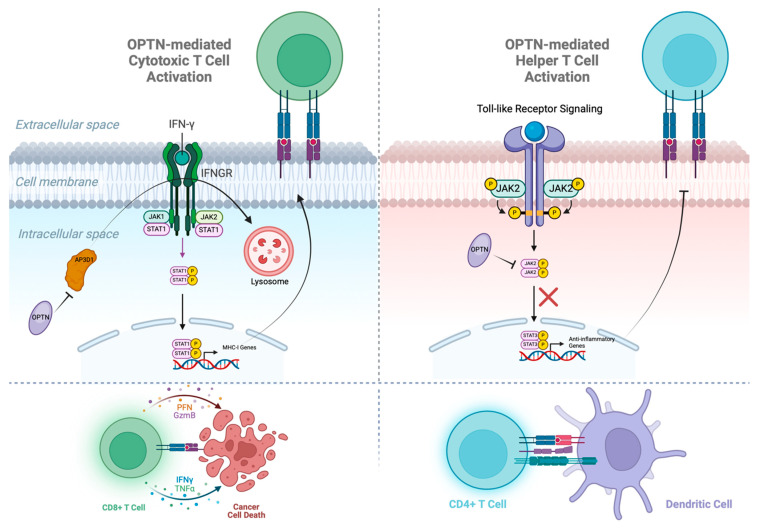
OPTN assists in activating both cytotoxic and helper T cells. In cancer cells, OPTN blocks the premature degradation of IFNGR via vesicular trafficking protein AP3D1, allowing the JAK1/STAT1 pathway to continue producing the MHC-1 receptors that activate cytotoxic T cells. In dendritic cells, OPTN blocks the JAK2/STAT3 pathway from translocating to the nucleus and transcribing anti-inflammatory genes, which limit the MHC-II receptors that activate helper T cells.

OPTN is emerging as a crucial immunological target due to its role in modulating immune responses and its potential to overcome resistance to immune therapies (Figure 3). Recent studies indicate that increased OPTN expression correlates with higher clinical benefit rates in patients receiving immune checkpoint blockade therapy. OPTN protects against cancer immune evasion by playing a pivotal role in maintaining IFN-γ-mediated anti-tumor immune surveillance in CRC. During CRC progression from adenoma to invasive carcinoma, OPTN expression is gradually lost, which is associated with an increased ability of cancer cells to evade immune detection [8]. The IFN-gamma/STAT1/IRF1 axis is essential for MHC-I antigen presentation, which allows cytotoxic T cells to recognize and eliminate tumor cells [35]. In vivo studies demonstrate that OPTN-deficient CRC cells exhibit enhanced tumor growth in immunocompetent mice, but not in immunodeficient models, highlighting its role in immune-mediated tumor control [8].

At the molecular level, IFN-γ binds to its receptor, IFNGR1, leading to the dimerization and nuclear translocation of STAT1, which subsequently induces the transcription of IRF1 [36]. IRF1, in turn, upregulates MHC-I gene expression, ensuring the presentation of tumor antigens to T cells [36]. However, the loss of OPTN results in the premature degradation of IFNGR1, impairing this critical signaling cascade [8]. The underlying mechanism involves lysosomal sorting of IFNGR1 mediated by AP3D1, a vesicular trafficking protein. Normally, IFNGR1 is stabilized on the cell membrane, but in the absence of OPTN, AP3D1 binds to palmitoylated IFNGR1 (Cys-122) and directs it towards lysosomal degradation [36]. OPTN prevents this by competitively binding to AP3D1, thereby preserving IFNGR1 stability and sustaining IFN-gamma signaling [8]. Consequently, loss of OPTN leads to reduced antigen presentation, impaired T cell activation, and immune evasion [8]. These findings position OPTN as a potential therapeutic target for enhancing immunotherapy efficacy in resistant cancers.

OPTN has also been identified as a crucial regulator of Dendritic cell (DC) maturation and function [9]. DCs serve as the most potent antigen-presenting cells (APCs), bridging innate and adaptive immune responses. Their immunostimulatory potential is contingent upon their maturation status. While immature DCs secrete anti-inflammatory cytokines, suppressing immune activation, mature DCs migrate to lymph nodes to prime T cells and orchestrate robust immune responses [37,38]. Within bone marrow-derived dendritic cells, OPTN deficiency skews their cytokine secretion profile towards an anti-inflammatory state. Specifically, OPTN-deficient DCs exhibit increased secretion of IL-10, while producing significantly lower levels of pro-inflammatory Th1 (IFN-gamma) and Th17 cytokines. This results in impaired CD4+ T cell proliferation and weaker antitumor immunity. Mechanistically, OPTN negatively regulates the JAK2/STAT3 signaling pathway, which governs IL-10 production and DC differentiation. OPTN inhibits this pathway by binding to the JH1 domain of JAK2, preventing its dimerization and subsequent activation of STAT3 [9]. Notably, this function is independent of OPTN’s UBD and its role in autophagy, establishing a novel immune-regulatory function for OPTN.

## 7. Therapeutic Implications of OPTN in Cancer

Given its diverse cellular roles as a multifunctional regulator in autophagy, vesicular trafficking, and immune signaling, OPTN represents a compelling yet complex target in cancer therapy. In particular, tumors characterized by OPTN overexpression or tumor-promoting activity may be especially responsive to therapeutic interventions aimed at modulating its function. However, the pleiotropic and context-dependent nature of OPTN introduces significant challenges for clinical targeting.

First, loss of OPTN disrupts LC3-II formation, autophagosome assembly, and cellular stress tolerance, therefore inhibiting OPTN function may elicit cytotoxic effects in normal tissues [4,7,27]. OPTN is broadly expressed in critical organs such as the brain, retina, and heart, and pathogenic mutations in the gene have been linked to neurodegenerative conditions, including glaucoma and amyotrophic lateral sclerosis (ALS) [1,30]. Therefore, systemic inhibition of OPTN carries considerable risks, including neurotoxicity and tissue degeneration. Moreover, OPTN depletion promotes IFNγ receptor 1 degradation through AP3D1, thereby suppressing IFNγ/MHC-I signaling and weakening T-cell-mediated immunity; such effects could undermine the therapeutic efficacy of immune checkpoint blockade [8]. Adding further complexity, OPTN exhibits functional redundancy with other autophagy receptors, including SQSTM1, Nuclear dot protein 52 (NDP52), and Tax1-binding protein 1 (TAX1BP1) [31,32]. Consequently, inhibition of OPTN may inadvertently activate compensatory survival pathways rather than induce tumor cell death. Finally, from a pharmacological standpoint, OPTN poses an additional challenge because it lacks a defined catalytic site and exerts its effects largely through protein–protein interactions involving its UBAN, LIR, and coiled-coil domains [2]. These structural features make selective drug design inherently difficult.

Nonetheless, efforts have been made to manipulate OPTN levels pharmacologically. Saikosaponin D (SSD), a triterpenoid saponin derived from Bupleurum falcatum L., has been investigated for its ability to degrade OPTN [39]. Cycloheximide chase and Western blot analyses demonstrated that SSD enhances OPTN degradation, leading to enhanced JAK2/STAT3 signaling and increased IL-10 production in DCs. These findings suggest that pharmacological manipulation of OPTN may indirectly reprogram immune responses within the tumor microenvironment. However, despite its emerging importance, clinical exploration of OPTN remains limited, partly due to the absence of clinically approved drugs targeting OPTN directly. Although SSD has been identified as a natural compound that destabilizes OPTN, it is not a specific OPTN inhibitor, highlighting the need for more precise pharmacological tools to modulate OPTN activity.

## 8. Conclusions and Future Directions

Taken together, these observations highlight that while OPTN represents a biologically significant and potentially druggable target in cancer, its multifaceted physiological functions demand extreme caution in therapeutic targeting. Off-target toxicity, impaired immune competence, compensatory resistance, tumor-specific variability, and unavailability of specific inhibitors remain key concerns in its clinical translation [40]. Still, the advent of artificial intelligence (AI)-driven drug discovery has reinvigorated the search for small-molecule inhibitors targeting proteins once deemed “undruggable” [41]. Machine learning algorithms can now facilitate de novo ligand design and predict binding interactions within non-catalytic domains, such as those present in OPTN [42]. Developing individual domain-specific inhibitors can mitigate potential side effects associated with targeting OPTN as a whole by preserving its other functional interactions. Complementing this, emerging therapeutic modalities, such as Proteolysis-Targeting Chimeras (PROTACs), RNA-based therapeutics, and Clustered regularly interspaced short palindromic repeats (CRISPR)-Cas9 gene editing, may offer innovative avenues to precisely manipulate OPTN expression or stability in cancer cells [43].

Additionally, to mitigate systemic side effects, a tumor-specific approach to OPTN inhibition may be considered. Recent advances in nanotechnology-based delivery systems provide promising solutions in this regard. Platforms such as lipid nanoparticles and polymeric nanoparticles enable the encapsulation and targeted delivery of small molecules, RNA therapeutics, or gene-editing tools directly to the tumor site. These systems are gaining traction in oncology due to their structural flexibility, biocompatibility, and capacity to enhance therapeutic precision while reducing systemic toxicity [44,45,46]. Targeting OPTN specifically within the tumor microenvironment, using these advanced delivery systems, may allow researchers to harness its therapeutic potential without compromising normal tissue function. Looking forward, the integration of OPTN-targeted interventions with biomarker-guided patient selection and rational combination strategies could transform OPTN from a biologically intriguing protein into a clinically actionable cancer target. Continued multidisciplinary efforts spanning molecular biology, pharmacology, and nanomedicine will be essential to realize this potential.

## Data Availability

No new data were created or analyzed in this study.
